# Gender and age disparities in attitude and preferences of probiotic and prebiotic-rich foods and supplements among adults in Saudi Arabia

**DOI:** 10.3389/fpubh.2026.1844860

**Published:** 2026-05-29

**Authors:** Rouba Khalil Naaman, Wejdan Talal Alghafari

**Affiliations:** Department of Clinical Nutrition, Faculty of Applied Medical Sciences, King Abdulaziz University, Jeddah, Saudi Arabia

**Keywords:** age, attitudes, gender, prebiotics, preferences, probiotics, Saudi Arabia

## Abstract

**Introduction:**

In recent decades, probiotic and prebiotic-rich foods and supplements have gained considerable attention for their health benefits, particularly the promotion of gut health. Despite the widespread publicity around these benefits, there is a lack of recognition of the effects of gender and age on attitudes and preferences regarding the intake of these foods and supplements. Therefore, this study aimed to assess gender and age differences in attitudes and preferences concerning probiotic and prebiotic-rich foods and supplements among adults in Saudi Arabia.

**Methods:**

A cross-sectional study was conducted between May and July 2023 among adults aged ≥18 years recruited from different regions of Saudi Arabia. An online valid questionnaire was used to assess attitudes and preferences regarding probiotic and prebiotic-rich foods and supplements.

**Results:**

A total of 915 participants completed the survey. Probiotics and prebiotics ever users, and never users accounted for 62, and 38% of the participants, respectively. Among the ever users, the main reason for using probiotics and prebiotics was gastrointestinal problems (57%). Females were more likely than males to prefer online or international pharmacies as sources of probiotics and prebiotics (24% vs. 12%, *p* = 0.003). Younger participants were more likely to get probiotic and prebiotic supplements from online or international pharmacies compared to older participants (*p* = 0.001). Among the never users, males were more likely than females to use probiotics and prebiotics in the future if a health professional recommended them (60% vs. 40%, *p* = 0.002).

**Discussion/conclusion:**

Gender and age play a role in adults’ attitudes and preferences concerning probiotic and prebiotic-rich foods and supplements, particularly in terms of preferred sources as well as the forms and duration of consumption. Further studies are required to examine more determinants of probiotic and prebiotic intake.

## 1 Introduction

Over the past few decades, probiotics and prebiotics have gained considerable attention for their potential health benefits, particularly in the area of gut health promotion. Probiotics are live microorganisms that, when consumed in adequate amounts, confer health benefits to the host (1). Prebiotics are nondigestible food components that promote the growth of beneficial bacteria in the gut (2). Their use has been linked to several health benefits, including the treatment of digestive disorders, such as diarrhea related to antibiotic use, constipation, irritable bowel syndrome, and inflammatory bowel diseases (3). Probiotics and prebiotics may boost the immune system (4). Their supplementation can lower serum cholesterol, prevent atopic allergies and cancer, and reduce hypertension (5).

Moreover, probiotics and prebiotics play a significant role in maintaining the balance of the gut microbiome and regulating metabolic, hormonal, immune, and inflammatory responses (6). The gut–brain axis, a complex bidirectional communication network linking the gastrointestinal tract and the central nervous system, is strongly modulated by the gut microbiome (7). This modulation occurs through the influence of the microbiome on neurotransmitter synthesis and metabolism (8), thereby affecting brain function and the regulation of stress and anxiety (9). Previous studies have demonstrated a positive association between maintaining a balanced microbiome, supporting the gut–brain axis, and promoting overall health (10–12).

Probiotics are commonly found in fermented foods and supplements. Yogurt was the first food to which probiotics were added (13). Today, many other foods contain probiotics, including cheese, milk, and ice cream (14). Nondairy probiotics, such as kombucha tea, herbal tea, baking mixes, and cereal-based products, have also become widespread as alternative sources of probiotics (14). Still, more clinical studies examining the exact probiotic effect on humans are needed to substantiate any health-promoting claims associated with these products (15). There are also a variety of probiotic supplements on the market, including liquids, powders, pills, capsules, tablets, and gelcaps (16). According to market analysis, probiotic supplements and foods (e.g., dairy products) are the main drivers of the functional foods industry, which reflects their popularity (17). Prebiotics are naturally present in various plant foods, including fruits, vegetables, whole grains, and legumes.

Globally, consumers are becoming increasingly conscious of the connection between diet and well-being; as a result, they look for safe and healthful products in addition to those that are tasty and appealing (18). The use of probiotics and prebiotics has evolved over the past decade. The evidence on the use of probiotics varies greatly among studies because different populations have been investigated, and different definitions of probiotic use have been proposed. For example, Nguyen et al. (19) found that probiotic use was prevalent in their sample (39.4% of their surveyed participants), but the majority of individuals were unaware of the supplement. A study that assessed the use of probiotics in New Zealand reported a prevalence of 25% (20). Other studies have examined attitudes and preferences concerning probiotic and prebiotic-rich foods and supplements (21). One study investigated university students’ attitudes and behaviors toward probiotic and prebiotic sources and supplements and found that the most common reason for consuming the foods in question was to strengthen the immune system (22). Another study explored adults’ attitudes toward gut health and their use of probiotics and prebiotics and reported that the primary motivation for consuming them was to preserve their health (23).

Despite the recognized benefits of probiotics and prebiotics, few studies have investigated the factors influencing their intake. As is the case with other nutrients, the consumption of probiotics and prebiotics could be influenced by several factors that might be associated with health outcomes. It has been reported that individuals’ attitudes and preferences concerning food and supplements can be affected by a number of elements, such as physiological, psychological, environmental, cognitive, and sociodemographic factors (24). Among the latter, gender and age are particularly relevant (25). Gender differences influence the perception of healthy foods, with females focusing more on nutritional value and healthy eating than males. It has also been reported that women are more likely to avoid eating red meat, white flour, and food additives and more likely to eat vegetables, fruit, fish, and dairy products compared to men (25). Furthermore, many scholars have found that age influences food preferences (26). In one study, younger individuals preferred snacks, including potato chips, ice cream, and cookies, while individuals over 55 were more likely to prefer meals such as soup, burgers, and side dishes (27). A better understanding of the effects of gender and age-related food preferences is crucial for the development of effective strategies to enhance the intake of probiotics and prebiotics, which are associated with managing and preventing diet-related diseases.

In Saudi Arabia, few studies have investigated consumers’ knowledge, attitudes, and preferences, as well as associated factors, concerning the intake of probiotics and prebiotics. Faden et al. (28) assessed the public’s knowledge, awareness, and attitudes regarding probiotic intake and found variations in people’s levels of knowledge of this nutrient. A high level of knowledge was reported in a recent study that aimed to assess awareness, knowledge, and beliefs regarding prebiotics and probiotics in people living in Saudi Arabia (29). Other studies have explored the knowledge, practices, and attitudes concerning probiotics of Saudi health science students and healthcare providers (30, 31). However, there are no studies on the factors that can affect attitudes and preferences concerning probiotic and prebiotic foods and supplements; this is particularly the case with gender and age.

Understanding the use patterns of these foods and supplements, as well as their influencing factors, is essential for promoting their integration into Saudi dietary practices. Therefore, this study aimed to assess the effects of gender and age on attitudes and preferences regarding probiotic and prebiotic-rich foods and supplements among adults living in Saudi Arabia.

## 2 Materials and methods

### 2.1 Study design

Approval for this cross-sectional study was obtained from the Unit of Biomedical Research Ethics Committee at King Abdulaziz University, Jeddah, Saudi Arabia (reference no. 554–22). The participants provided their consent at the start of the online questionnaire.

### 2.2 Participants and recruitment

Recruitment took place between May and July 2023. An online questionnaire was created using Google Forms, and its link was shared on social media platforms, including WhatsApp and X (formerly known as Twitter). Individuals were eligible to participate if they were aged ≥18 and residents of Saudi Arabia.

### 2.3 The questionnaire

The questionnaire used was developed for this study to assess attitudes and preferences concerning probiotics and prebiotics among adults living in Saudi Arabia. It was based on the attitude and preferences section of a questionnaire published by Khalesi et al. (23). Several changes were made to the latter, including modifying some options and removing questions that were irrelevant to the aim of the current study. The questionnaire was then translated into Arabic using Brislin’s back-translation method (32). One gastroenterologist and four nutrition specialists, including four PhD holders and one MSc holder, tested the questionnaire’s clarity and ease of administration. Some questions and options were edited and reformulated. The final questionnaire was subjected to tests of internal consistency, reliability, and factor analysis (exploratory and confirmatory) to confirm construct validity in a pilot study involving 25 volunteers. A Cronbach’s alpha value of 0.72 was achieved, while an average coefficient of correlation of 0.81 was obtained between the test and re-test groups. Bartlett’s test of sphericity indicated that the correlation matrix was not random, *p* = 0.008, while the Kaiser–Meyer–Olkin measure of sampling adequacy was 0.83. The final version of the questionnaire comprised two main sections, and it had a completion duration of 5–7 min. Before answering the questionnaire, the participants were made aware of the study’s aims and inclusion criteria, as well as the fact that the data would be kept confidential (see Supplementary File 1 for the English language version of the questionnaire).

In the first part of the questionnaire, the participants were required to provide their sociodemographic and background information, including age, gender, marital status, nationality, place of residence, education level, current work status, monthly income, field of study, history of chronic diseases, and smoking status. They were also asked to self-report their height in centimeters and weight in kilograms, which were used to obtain their body mass index (BMI) values.

The second part of the questionnaire assessed attitudes and preferences concerning probiotics and prebiotics. The participants were asked whether they were taking any type of probiotics or prebiotics from foods or supplements by selecting one of the following answers: “yes,” “used to take them as supplements,” and “no.” The participants who responded “yes” were classified as current users, while those who selected “used to take them as supplements” were classified as past users (i.e., individuals who had previously used probiotics/prebiotics but were not using them at the time of the survey); both groups were subsequently combined and classified as “ever users” for analysis. Those who answered yes had to answer several other questions related to the frequency, reasons, and main influences of their consumption. They were also instructed to choose their preferred sources of consumption, including natural foods, supplements bought at local pharmacies, supplements bought from online/international pharmacies, and no preference. Information was also collected regarding the preferred forms of supplementation. The participants who answered “used to take them as supplements” were asked about the reasons, periods, and forms of their consumption. They were also asked why they had stopped taking such supplements. The participants who answered “no” were asked about their reasons for not consuming probiotics and prebiotics, their preferred sources of these foods, and whether they would be willing to use probiotics and prebiotics in the future.

### 2.4 Sample size calculation

The Raosoft software was used to calculate the sample size. In order to estimate a prevalence of 50% of supplements use within a 5% margin of error at a 95% confidence interval, a minimum sample size of 385 participants are needed. In these calculations the total population of adults living in Saudi Arabia in 2019 was estimated at 23,468,225, as per the Saudi General Authority for Statistics (33).

### 2.5 Statistical analysis

The data were analyzed using SPSS Statistics version 28 (IBM, Armonk, NY, United States). The data are reported as numbers and percentages. The association between categorical variables was tested using the chi-square test. Multivariable binary logistic regression analysis was performed to assess the independent determinants of probiotic and prebiotic preferred forms, sources, and willingness to use in the future. A *p*-value of <0.05 was considered statistically significant.

## 3 Results

### 3.1 Participants’ backgrounds

Table 1 shows the demographic characteristics of the study population. A total of 915 participants completed the survey. Ever users and never users of probiotics and prebiotics accounted for 62 and 38% of the sample, respectively. Of the participants, 44% were aged 18–24 years and 28% were aged ≥40. Female participants accounted for 72% of the study population. Most of them were Saudis (93%) and came from the western region (45%). Fifty-nine of the respondents had university degrees. The most common (29%) reported income class was >10,000 Saudi Riyals (>$2,600) per month. Forty-three percent of the participants worked in a scientific field. Only 20% had chronic illnesses. Nonsmokers represented 86% of the sample. Normal-weight and overweight respondents made up 43 and 31% of the sample, respectively. There were significant differences in the distribution of the age groups (*p* = 0.010), region (*p* = 0.005), work status (*p* = 0.023), history of chronic diseases (*p* = 0.009), and BMI (*p* = 0.044).

**Table 1:** Demographic and background characteristics of the participants (*n* = 915).

Variables	Total (*N* = 915)	Probiotics and prebiotics using groups		*p*-value
		Ever users (*N* = 570)	Never users (*N* = 345)	
Age				
18–24	395 (44)	229 (40)	166 (48)	0.010
25–39	260 (28)	160 (28)	100 (29)	
≥40	260 (28)	181 (32)	79 (23)	
Gender				
Male	253 (28)	151 (26)	102 (30)	0.314
Female	662 (72)	419 (74)	243 (70)	
Marital status				
Single	495 (54)	292 (52)	203 (59)	0.156
Married	386 (42)	256 (44)	130 (38)	
Widowed	10 (1)	7 (1)	3 (1)	
Divorced	24 (3)	15 (3)	9 (2)	
Nationality				
Saudi	849 (93)	532 (93)	317 (92)	0.411
Non Saudi	66 (7)	38 (7)	28 (8)	
Region				
Western region	415 (45)	245 (43)	170 (49)	0.005
Central region	208 (23)	136 (24)	72 (21)	
Eastern region	178 (19)	128 (22)	50 (15)	
Northern region	27 (3)	17 (3)	10 (3)	
Southern region	87 (10)	44 (8)	43 (12)	
Educational level				
Less than high school	26 (3)	17 (3)	9 (3)	0.474
High school	245 (27)	154 (27)	91 (26)	
University level	539 (59)	327 (57)	212 (61)	
Higher degrees	105 (11)	72 (13)	33 (10)	
Work status				
Student	347 (38)	206 (36)	141 (41)	0.023
Employed	283 (31)	173 (30)	110 (32)	
Unemployed	169 (18)	103 (18)	66 (19)	
Retired	74 (8)	54 (10)	20 (6)	
Business/trading	42 (5)	34 (6)	8 (2)	
Income				
No income	150 (16)	81 (14)	69 (20)	0.142
<2,000 SR	260 (29)	162 (28)	98 (28)	
2000–4,000 SR	104 (11)	73 (13)	31 (9)	
5,000–7,000 SR	67 (7)	39 (7)	28 (8)	
8,000–10,000 SR	69 (8)	45 (8)	24 (7)	
>10,000 SR	265 (29)	170 (30)	95 (28)	
Field of study				
Medical	215 (23)	136 (24)	79 (23)	0.272
Scientific	390 (43)	233 (41)	157 (46)	
Literature	244 (27)	163 (28)	81 (2)	
No specific field	66 (7)	38 (7)	28 (8)	
History of chronic illness				
No	731 (80)	440 (77)	291 (84)	0.009
Yes	184 (20)	130 (23)	54 (16)	
Smoking				
No	790 (86)	495 (87)	295 (86)	0.636
Yes	93 (10)	54 (9)	39 (11)	
Ex-smoker	32 (4)	21 (4)	11 (3)	
Body mass index (BMI)	*N* = 910	*N* = 567	*N* = 343	
Underweight	74 (8)	35 (6)	39 (11)	0.044
Normal	393 (43)	247 (44)	146 (43)	
Overweight	278 (31)	181 (32)	97 (28)	
Obese	165 (18)	104 (18)	61 (18)	

Data are presented as number and percentage. Differences were assessed by Chi-square test. BMI was calculated by using self-reported weight and height, data missing for 5 participants and was classified based on the centers for disease control and prevention categories (62).

### 3.2 Attitudes and preferences concerning probiotics and prebiotics

#### 3.2.1 Ever users

Gastrointestinal problems (57%) and maintaining good health and well-being (32%) were the main reasons for using probiotics and prebiotics. Regarding supplements, capsules, and tablets were the preferred forms (41%) (Table 2). Regarding the forms of supplements, liquids were consumed more often by males than females (29% vs. 17%, *p* = 0.001), while capsules and tablets were taken more often by females than males (43% vs. 35%, *p* = 0.081).

**Table 2:** Attitude and preference of probiotics and prebiotics ever users.

Attitude and preferences	Total		Gender			Age (years)			
	*n*	%	Male (*N* = 151)	Female (*N* = 419)	*p*-value	18–24 (*N* = 229)	25–39 (*N* = 160)	≥40 (*N* = 181)	*p*-value
Ever users (current and past users) (*n* = 570)									
Main reason for using probiotics and prebiotics									
Gastrointestinal discomforts	317	57	90 (61)	277 (55)	0.483	119 (53)	86 (54)	112 (63)	0.377
Immune system issues	12	2	2 (1)	10 (2)		8 (4)	3 (2)	1 (1)	
Replenish friendly bacteria when taking antibiotics	23	4	7 (4)	16 (4)		9 (4)	8 (5)	6 (3)	
To maintain health and wellbeing	180	32	45 (31)	135 (33)		76 (34)	52 (33)	52 (30)	
Others	27	5	4 (3)	23 (6)		12 (5)	9 (6)	6 (3)	
Form of taking supplements									
Liquid	112	20	43 (29)	69 (17)	0.001	38 (17)	36 (23)	38 (21)	0.300
Capsule or tablet	230	41	51 (35)	179 (43)	0.081	84 (37)	73 (46)	73 (41)	0.215
Powder	41	7	12 (8)	29 (7)	0.627	19 (8)	9 (6)	13 (7)	0.601
Current users (*n* = 499)									
Frequency of taking pro/prebiotics									
3–7 times a week	216	43	65 (50)	151 (41)	0.233	87 (42)	58 (44)	71 (44)	0.973
At least once a week	233	47	54 (41)	179 (49)		97 (47)	61 (46)	75 (47)	
At least once a month	50	10	12 (9)	38 (10)		22 (11)	14 (10)	14 (9)	
Main influence to try probiotics and prebiotics									
My own general health knowledge and beliefs	338	68	93 (71)	245 (67)	0.235	134 (65)	98 (74)	106 (66)	0.162
Health professionals	49	10	11 (8)	38 (10)		15 (7)	15 (11)	19 (12)	
Friends and family	70	14	21 (16)	49 (14)		36 (18)	13 (10)	21 (13)	
Advertisements or social media	42	8	6 (5)	36 (9)		21 (10)	7 (5)	14 (9)	
Preferred source of probiotics and prebiotics									
Natural food sources	395	79	101 (77)	294 (80)	0.499	163 (71)	105 (66)	127 (70)	0.483
Local pharmacy supplements	72	14	20 (15)	52 (14)	0.751	28 (12)	18 (11)	25 (14)	0.767
Online/international pharmacy supplements	106	21	16 (12)	90 (24)	0.003	51 (22)	37 (23)	18 (10)	0.001
No preference	19	4	8 (6)	11 (3)	0.109	9 (4)	4 (2)	7 (4)	0.715
Past users (*n* = 71)									
Duration of using probiotics and prebiotics supplements									
<1 month	41	57	12 (60)	29 (56)	0.001	14 (61)	15 (56)	12 (54)	0.535
1–3 months	17	24	1 (5)	16 (31)		5 (22)	5 (19)	7 (32)	
3–6 months	5	7	0 (0)	5 (9)		3 (13)	2 (7)	0 (0)	
6–12 months	5	7	3 (15)	2 (4)		1 (4)	2 (7)	2 (9)	
>1 year	4	5	4 (20)	0 (0)		0 (0)	3 (11)	1 (5)	
Main reason for stopping taking probiotics and prebiotics									
Cost	8	11	3 (15)	5 (10)	0.731	2 (8)	1 (4)	5 (23)	0.142
Side effects	8	11	2 (10)	6 (11)		2 (8)	6 (22)	0 (0)	
Low effectiveness	4	6	0 (0)	4 (8)		0 (0)	2 (7)	2 (9)	
No concerns/issues in consuming these products any more	23	32	7 (35)	16 (31)		8 (35)	8 (30)	7 (32)	
Others	29	40	8 (40)	21 (40)		11 (49)	10 (37)	8 (36)	

Data are presented as number and percentage. Differences were assessed by Chi-square test. Form of taking supplements and preferred source of probiotics and prebiotics: not mutual exclusive (i.e sum exceeded 100%).

About half (47%) of current probiotic and prebiotic users reported an intake of at least once a week, whereas 43% reported an intake of 3–7 times a week. The main influence on trying probiotics and prebiotics was the participants’ general health knowledge and beliefs (68%). Natural foods were the preferred source of probiotics and prebiotics (79%). Females were more likely to obtain probiotic and prebiotic supplements from online or international pharmacies than males (24% vs. 12%, *p* = 0.003) (Table 2). Participants aged 25–39 were more likely to get probiotic and prebiotic supplements from online/international pharmacies (*p* = 0.001) (Table 2).

Regarding the past users, the duration of intake was less than 1 month for 57% of the participants, and it exceeded 1 year only among 5% of them. The main reasons for no longer consuming probiotics and prebiotics were no concerns or issues in consuming these products (32%) and other motives, such as unavailability on the market, unpleasant taste, improved health, the desire to use them only when needed, and a changed lifestyle (40%). There was a statistically significant difference between males and females regarding the duration of probiotic and prebiotic supplement use. Specifically, 60% of men and 56% of women consumed them for less than 1 month, whereas 35% of males and only 4% of females used them for longer than 6 months (*p* = 0.001) (Table 2).

#### 3.2.2 Never users

The majority of never user participants were willing to consume probiotics and prebiotics in the future if their health professionals recommended them (46%) or if they became interested in trying them (43%). The most frequently reported reasons for not using probiotics and prebiotics were not believing that one needed them, given their good health (34%) and not knowing about them (34%). For never users, the preferred hypothetical source of probiotics and prebiotics was natural foods (69%) (Table 3).

**Table 3:** Attitude and preference of probiotics and prebiotics never users (*n* = 345).

Attitude and preferences	Total		Gender			Age (years)			
	*n*	%	Male (*N* = 102)	Female (*N* = 243)	*p*-value	18–24 (*N* = 166)	25–39 (*N* = 100)	≥40 (*N* = 79)	*p*-value
Willingness to use probiotics and prebiotics in future									
Yes, only if my health professional recommended	158	46	61 (60)	97 (40)	0.002	69 (42)	46 (46)	43 (54)	0.449
Yes, I would be interested in trying	150	43	31 (30)	119 (49)		77 (46)	42 (42)	31 (39)	
No, I’m not interested in trying	38	11	10 (10)	28 (11)		20 (12)	12 (12)	6 (7)	
Main reason for not using probiotics and prebiotics									
Cost	34	10	15 (15)	19 (8)	0.180	18 (11)	8 (8)	8 (10)	0.715
Side effects	11	3	1 (1)	10 (4)		8 (5)	1 (1)	2 (3)	
I do not believe in their effectiveness	13	4	5 (5)	8 (3)		5 (3)	6 (6)	2 (3)	
I am healthy; I do not believe I need them	117	34	34 (33)	83 (34)		58 (35)	33 (33)	26 (32)	
I do not know about probiotics and prebiotics	117	34	35 (34)	82 (34)		55 (33)	33 (33)	29 (36)	
I do not like supplements	54	15	12 (12)	42 (17)		22 (13)	19 (19)	13 (16)	
Preferred source of probiotics and prebiotics, if would be taken in the future									
Natural food sources	239	69	77 (75)	162 (66)	0.248	109 (66)	70 (70)	60 (75)	0.008
Supplements	60	17	14 (14)	46 (19)		36 (21)	9 (9)	15 (19)	
No preference	47	14	11 (11)	36 (15)		21 (13)	21 (21)	5 (6)	

Data were assessed as number and percentage. Differences were assessed by Chi-square test.

Males were more likely than females (60% vs. 40%) to use probiotics and prebiotics in the future if a health professional recommended them, while females were more likely than males (49% vs. 30%) to consume such foods if they became interested in trying them (*p* = 0.002) (Table 3).

Never users aged ≥40 years were the most likely to prefer natural food sources for probiotics and prebiotics in the future (75%), whereas 21% of those aged 18–24 years would prefer supplements compared to the other age groups (*p* = 0.008) (Table 3).

### 3.3 Factors associated with probiotic and prebiotic use and preferences

In the multivariable binary logistic regression analysis, gender, educational level, and income were significantly associated with supplement form preference among ever users. Male participants were more likely to prefer liquid forms (OR = 1.8, *p* = 0.025) and less likely to prefer capsules and tablets (OR = 0.5, *p* = 0.015). A higher educational level was associated with a lower likelihood of preferring liquid forms (OR = 0.3, *p* = 0.042). Income was significantly associated with preference for powder forms, particularly among participants with middle to lower income levels. Additionally, overweight participants were more likely to prefer liquid forms (OR = 2.3, *p* = 0.002). No significant associations were observed for age or history of chronic illness (*p* > 0.05) (Table 4).

**Table 4:** Multivariable binary logistic regression analysis of factors associated with preferred forms of probiotic and prebiotic supplements in ever users.

Variables	Form of supplements								
	Liquid			Capsule or tablet			Powder		
	OR	95% CI	*p*-value	OR	95% CI	*p*-value	OR	95% CI	*p*-value
Age									
25–39	1.4	0.7–2.9	0.254	1.3	0.8–2.3	0.241	0.7	0.2–2.0	0.558
≥40	0.8	0.3–1.7	0.604	1.1	0.6–2.1	0.577	0.7	0.2–2.3	0.684
Gender									
Male	1.8	1.0–3.0	0.025	0.5	0.3–0.8	0.015	1.6	0.7–3.7	0.249
Educational level									
Less than high School	1.2	0.3–3.9	0.747	0.5	0.1–1.7	0.328	4.4	0.9–20.9	0.057
University level	0.6	0.3–1.0	0.097	0.8	0.5–1.2	0.403	1.6	0.6–3.8	0.268
Higher degrees	0.3	0.1–0.9	0.042	0.9	0.4–1.9	0.970	1.6	0.3–6.6	0.510
Income									
No income	1.2	0.5–2.9	0.627	0.9	0.5–1.9	0.982	5.6	0.6–50.6	0.122
<2,000 SR	1.0	0.4–2.3	0.887	0.7	0.4–1.4	0.387	10.4	1.3–84.4	0.027
5,000–7,000 SR	1.6	0.6–4.4	0.299	0.9	0.4–2.0	0.855	10.9	1.1–99.9	0.034
8,000–10,000 SR	1.0	0.3–2.9	0.926	0.7	0.3–1.7	0.568	5.6	0.5–58.5	0.146
>10,000 SR	1.3	0.5–2.9	0.496	1.0	0.5–1.9	0.912	3.4	0.3–29.9	0.268
History of chronic illness									
Yes	1.1	0.6–1.9	0.649	0.9	0.6–1.5	0.994	1.0	0.4–2.4	0.899
Body mass index (BMI)									
Underweight	1.3	0.5–3.6	0.539	0.4	0.2–1.1	0.093	0.0	0.0–6.9	0.956
Overweight	2.3	1.3–4.0	0.002	0.8	0.5–1.3	0.631	1.3	0.5–2.9	0.494
Obese	1.7	0.8–3.3	0.106	1.2	0.7–2.1	0.384	1.0	0.3–2.9	0.883

OR, odd ratio; CI, confidence interval.

Among current users, age, gender, income, and history of chronic illness were significantly associated with preferred sources of probiotics and prebiotics. Participants aged ≥40 years had less likely to prefer online or international pharmacy sources (OR = 0.3, *p* = 0.015). Similarly, male participants were less likely to prefer online or international pharmacies compared to females (OR = 0.4, *p* = 0.036). Participants earning 5,000–7,000 SR were associated with a lower likelihood of preferring natural sources (OR = 0.3, *p* = 0.022). Participants with a history of chronic illness were significantly less likely to prefer natural food sources (OR = 0.4, *p* = 0.003) and more likely to prefer online or international pharmacy supplements (OR = 2.0, *p* = 0.020). No significant associations were found for educational level or BMI (*p* > 0.05) (Table 5).

**Table 5:** Multivariable binary logistic regression analysis of factors associated with preferred sources of probiotic and prebiotic in current users.

Variables	Preferred sources of probiotic and prebiotic											
	Natural food sources			Local pharmacy supplements			Online/International pharmacy supplements			No preference		
	OR	95% CI	*p*-value	OR	95% CI	*p*-value	OR	95% CI	*p*-value	OR	95% CI	*p*-value
Age												
25–39	1.0	0.5–2.1	0.791	0.7	0.3–1.7	0.523	1.2	0.6–2.3	0.537	0.4	0.0–2.4	0.353
≥40	1.5	0.7–3.3	0.245	0.6	0.2–1.5	0.363	0.3	0.1–0.8	0.015	0.6	0.1–3.4	0.636
Gender												
Male	0.7	0.4–1.3	0.385	0.9	0.4–1.8	0.877	0.4	0.2–0.9	0.036	1.9	0.6–6.2	0.244
Educational level												
Less than high school	2.9	0.3–25.6	0.316	1.0	0.2–5.2	0.963	0.0	0.0–3.4	0.956	0.0	0.0–0.0	0.973
University level	0.7	0.4–1.3	0.414	0.8	0.4–1.5	0.595	1.0	0.6–1.8	0.795	0.6	0.2–1.9	0.425
Higher degrees	1.3	0.5–3.4	0.576	0.5	0.1–1.4	0.215	1.1	0.4–2.8	0.835	1.1	0.2–6.8	0.847
Income												
No income	0.5	0.2–1.4	0.246	1.1	0.4–3.1	0.826	1.7	0.7–4.1	0.239	0.8	0.1–6.6	0.898
<2,000 SR	0.6	0.2–1.5	0.350	0.8	0.3–2.2	0.760	1.6	0.7–3.8	0.211	1.3	0.2–7.1	0.729
5,000–7,000 SR	0.3	0.1–0.8	0.022	1.8	0.5–5.6	0.309	2.6	0.9–7.5	0.066	0.0	0.0–1.2	0.959
8,000–10,000 SR	0.5	0.1–1.7	0.311	1.9	0.6–6.1	0.264	0.9	0.2–3.3	0.981	0.0	0.0–1.6	0.959
>10,000 SR	0.7	0.2–1.8	0.535	1.4	0.5–3.8	0.477	1.9	0.7–4.8	0.143	1.1	0.1–7.2	0.909
History of chronic illness												
Yes	0.4	0.2–0.7	0.003	1.7	0.9–3.2	0.077	2.0	1.1–3.6	0.020	0.5	0.1–2.0	0.377
Body mass index (BMI)												
Underweight	1.1	0.4–3.0	0.811	0.9	0.2–2.9	0.888	0.6	0.2–1.6	0.346	0.0	0.0–8.0	0.959
Overweight	0.8	0.5–1.5	0.644	1.4	0.7–2.7	0.220	0.8	0.4–1.4	0.475	1.1	0.3–3.8	0.881
Obese	0.9	0.4–1.8	0.851	0.9	0.4–2.1	0.973	0.6	0.3–1.4	0.299	2.9	0.8–9.8	0.086

OR, odd ratio; CI, confidence interval.

Among never users, gender was significantly associated with willingness to consume probiotics and prebiotics in the future. Male participants were more likely to be willing to consume these products if recommended by a health professional (OR = 2.1, *p* = 0.003), whereas females were more likely than males to consume them if they became interested in trying them (OR = 0.4, *p* = 0.004). No significant associations were found with other variables (*p* > 0.05) (Table 6).

**Table 6:** Multivariable binary logistic regression analysis of factors associated with willingness to use probiotics and prebiotics in future in never users.

Variables	Willingness to use probiotics and prebiotics in future								
	Yes, only if my health professional recommended			Yes, I would be interested in trying			No, I’m not interested in trying		
	OR	95% CI	*p*-value	OR	95% CI	*p*-value	OR	95% CI	*p*-value
Age									
25–39	0.8	0.4–1.6	0.557	1.1	0.6–2.3	0.598	1.1	0.4–2.9	0.842
≥40	0.9	0.4–2.1	0.927	1.1	0.5–2.5	0.697	0.7	0.2–2.6	0.669
Gender									
Male	2.1	1.2–3.6	0.003	0.4	0.2–0.7	0.004	0.9	0.4–2.2	0.913
Educational level									
Less than high School	4.6	0.8–25.9	0.083	0.3	0.0–2.0	0.250	0.0	0.0–2.2	0.967
University level	0.8	0.4–1.4	0.568	1.0	0.6–1.8	0.777	1.2	0.5–2.7	0.667
Higher degrees	0.9	0.3–2.4	0.886	0.9	0.3–2.4	0.894	1.2	0.2–6.2	0.748
Income									
No income	0.8	0.3–2.0	0.710	1.1	0.4–2.8	0.747	1.0	0.3–3.5	0.912
<2,000 SR	0.8	0.3–2.1	0.783	1.4	0.6–3.5	0.369	0.5	0.1–1.9	0.351
5,000–7,000 SR	1.6	0.5–4.8	0.396	0.9	0.3–2.8	0.907	0.3	0.0–2.1	0.265
8,000–10,000 SR	0.7	0.2–2.2	0.550	1.6	0.5–5.0	0.403	0.7	0.1–3.8	0.754
>10,000 SR	1.4	0.5–3.5	0.459	0.9	0.3–2.4	0.938	0.5	0.1–1.9	0.321
History of chronic illness									
Yes	0.6	0.3–1.2	0.177	1.5	0.8–2.9	0.145	0.8	0.3–2.4	0.791
Body mass index (BMI)									
Underweight	0.8	0.3–1.8	0.709	1.0	0.5–2.2	0.877	1.2	0.3–3.6	0.735
Overweight	1.1	0.6–2.0	0.530	0.7	0.4–1.2	0.258	1.3	0.5–3.1	0.451
Obese	1.3	0.8–3.3	0.442	0.9	0.4–1.7	0.776	0.5	0.1–1.9	0.389

OR, odd ratio; CI, confidence interval.

## 4 Discussion

This study assessed gender and age differences in attitudes and preferences concerning probiotic and prebiotic-rich foods and supplements among adults in Saudi Arabia. Its findings will help to make recommendations for informed and personalized advice by healthcare providers and tailor marketing strategies to consumers’ preferences. More than half of the participants used probiotics and prebiotics. Overall, significant differences were found between genders and across age groups regarding their preferred sources, forms, and duration of consumption of probiotic and prebiotic-rich foods and supplements.

Almost half of the users reported taking probiotics and prebiotics at least 3–7 times a week, with no significant differences between males and females or across age groups. While some studies have indicated that probiotics should be taken daily to be effective (34, 35), some have found a lack of consensus on the daily intake of probiotics and prebiotics (36). In an Australian study, 53.3% of participants consumed probiotics three times a month to five times a week (23). In the present study, the main reasons given for the consumption of probiotic and prebiotic foods and supplements were helping with gastrointestinal problems and maintaining good health and well-being. In the United States, the main reason for probiotic intake was to improve overall health and longevity (54%) as well as gastrointestinal symptoms, particularly bloating (45%) (37). In Australia, the main reported reasons were maintaining good health, alleviating gastrointestinal symptoms, and boosting immunity (23).

Among users, the main driver in trying probiotics and prebiotics was the participants’ general health knowledge and beliefs. Other studies have found that adults who pursue healthier lifestyles and have considerable knowledge of the advantages of probiotics and prebiotics are more likely to consume them than other individuals (36, 38, 39).

Many scholars have reported gender differences in probiotic and prebiotic consumption, with females being more likely to use them than males (23, 40, 41). This pattern has been attributed to higher health awareness and greater engagement in preventive health behaviors among women (40, 42). However, the present study did not find a significant difference in the use of probiotics and prebiotics between males and females. This finding may be influenced by the demographic composition of the sample, which was predominantly female, and thus may have reduced variability between groups and limited the ability to detect gender-based differences.

As expected, the present study found that females were more likely to report a preference for online or international pharmacies as sources of probiotics and prebiotics compared to males. It has previously been reported that women are more likely than men to use the internet to search for medical or health-related information (43). Moreover, a study discovered that online dietary supplement shopping was commonly practiced by Saudi women (44). This is consistent with a Swedish study that reported that female gender was a strong predictor of online medication shopping (45). This could be attributed to the fact that women have stronger positive beliefs concerning the benefits of internet-based health searches (46), in addition to their overall higher medication use compared to men (47).

Our findings show that male participants reported significantly higher consumption of liquid supplements than females, while females reported significantly higher consumption of tablets and capsules. Consistent findings were reported in an Iraqi study, which showed that the capsule form was preferred more by female participants than males (48). This could be explained by the fact that men might wish to easily ingest supplements, or they might mix them with other supplements in shakes (49, 50), whereas women might prefer tablets and capsules because they are tasteless (51). The current study found that participants with middle to lower incomes were more likely to prefer powder supplements. This may be explained by cost considerations, as powder formulations are generally more affordable than other supplement forms (52). These findings could be relevant to the probiotic industry, as they can help promote sustained use by current consumers and attract new consumers.

In terms of users’ ages, younger adults who constituted a large proportion of the current study sample were more likely than middle-aged and older adults to report a preference for online or international pharmacies as sources of probiotics and prebiotics. Similarly, a Saudi study on the use of dietary supplements among college students found that most of the participants who reported using such supplements preferred to purchase them from international stores (53). Online medicine shopping was also linked to younger age in another recent study (45). This result could be attributed to the amount of time young adults spend on online platforms, which might lead to a greater tendency toward online shopping and purchases compared to older adults (54). Additionally, the present study found that participants with a history of chronic illness were less likely to prefer natural food sources and more likely to prefer supplements obtained from online or international pharmacies. This preference can be explained as follows: individuals with chronic conditions may be more inclined to seek standardized or specialized supplements rather than through natural food sources (55).

Among this study’s past users, the main reasons for no longer using probiotics and prebiotics were a lack of continued interest in these products, unavailability in the market, unpleasant taste, improved health, the desire to use them only when needed, and lifestyle changes. Previous studies have reported similar barriers to upholding probiotic and prebiotic intake, including a lack of continued interest in these products, cost, unavailability, and unpleasant taste (23).

The present study revealed that female past users were more likely than male past users to report consuming probiotic and prebiotic supplements for short periods of time (1–3 months). However, a recent study that investigated the use of dietary supplements in middle-aged adults found that women took such supplements for longer periods than men (56). This discrepancy could be due to the large number of female participants in the present study. Moreover, in the present study, female past users primarily used probiotic and prebiotic supplements to manage gastrointestinal problems, and these supplements are often prescribed for a few weeks or months compared to other similar products (57).

The majority of never users, who were mainly male, stated that they would be willing to use probiotics and prebiotics in the future if their health professionals recommended them, whereas females stated that they would be willing to consume them if they became interested in trying them, which indicates a positive attitude toward these products. This emphasizes the role that healthcare professionals could play in promoting interest in and the utilization of probiotics and prebiotics. We could not explain the gender differences observed in this study; however, a biological factor might have been at play. Previous research from Australia (23) found that almost half of nonusers said that they would consume probiotics if recommended by their health professionals. This is contrary to what has been reported in the United Arab Emirates (58) and Australia (23), where most of the participants did not consider taking probiotics and prebiotics and were not even willing to learn about them.

In the present study, the most frequent reason for not using probiotics and prebiotics was the belief that there was no need to do so because the respondents were healthy and ignorant regarding these products. In another study, the most common reasons for not consuming probiotics were financial barriers and a positive health perception (59).

The present study found that almost two-thirds of never users would prefer natural food sources if they were to start taking these products in the future. A significant age difference was found among never users regarding the preferred sources of probiotics and prebiotics. Older adults reported a preference for natural food sources compared to younger adults, who preferred supplements. The former might have chosen natural foods due to traditional habits of whole food preference or as a result of polypharmacy, which might have made them wary of using dietary supplements. In contrast, younger adults have mixed preferences depending on their lifestyles, education levels, and health goals. Different findings have been reported by scholars who have assessed the influence of age on the consumption of dietary supplements and natural foods (60, 61).

The findings of this study should be employed with caution due to a number of limitations. First, due to the cross-sectional design, causal relationships between the findings cannot be established. Additionally, the study was not specifically powered to detect differences among subgroups (e.g., current, past, and non-users). Therefore, subgroup analyses should be interpreted with caution. Second, although collecting data online is easy and quick, the representativeness of this method is questionable, as only those familiar with the internet and online surveys will be included. This can affect the generalizability of the results. Third, the self-reporting nature of the study may have produced bias in the findings. In addition, the questionnaire lacked full definitions and food sources of probiotics and prebiotics, which might lead to misconceptions, and a limited understanding. The respondents who claimed to have never consumed probiotics and prebiotics might be incorrect, as many people may not be aware that prebiotics, in particular, are naturally present in commonly consumed food items. Even if they regularly consume these foods, they might not recognize them as sources of prebiotics. Fourth, most of the participants were women and were aged between 18 and 24 years, which might have influenced the results in several ways. This may have led to an overestimation of probiotic and prebiotic use, which is more common among females and younger individuals. In addition, gender and age may act as potential confounders of the observed associations, particularly in relation to supplement use patterns and source preferences. Finally, the findings may not fully reflect the behaviors and preferences of older adults and males in the general population.

## 5 Conclusion

Gender and age played a role in Saudi adults’ attitudes and preferences concerning probiotic and prebiotic-rich foods and supplements, particularly in their preferred sources, forms, and duration of consumption. Females and younger participants were more likely to prefer online or international pharmacies as sources of probiotics and prebiotics. The majority of male never users reported that they would be willing to use them in the future if their health professionals recommended them. Older never users strongly preferred natural food sources compared to younger never users, who preferred supplements to satisfy their needs for probiotics and prebiotics. The significant differences reported between genders and across age groups may help healthcare providers to educate the population about the health benefits of probiotics and prebiotics. Finally, the results of this study will allow food and dietary supplement manufacturers to tailor their products to consumers’ preferences.

## Data Availability

The raw data supporting the conclusions of this article will be made available by the authors, without undue reservation.
