# Supplementary material for: Gender and age disparities in attitude and preferences of probiotic and prebiotic-rich foods and supplements among adults in Saudi Arabia
Source: Front Public Health. 2026 May 29;14:1844860. doi: 10.3389/fpubh.2026.1844860 (PMC13260273; doi:10.3389/fpubh.2026.1844860)
Supplement: Supplementary file 1 [file Data_Sheet_1.DOCX]

Supplementary Material

**English language version of the questionnaire.**

**Additional file 1.**

| **Section 1: Sociodemographic and background section:** | | | |
| --- | --- | --- | --- |
| 1. How old are you?   ……………. (years) | | 1. Gender:  - Male - Female | |
| 1. What is your marital status?  - Single - Married - Divorced - Widowed | | 1. What is your nationality?  - Saudi - Non-Saudi | |
| 1. Place of residence:  - Western Region - Central Region - Eastern Region - Northern Region - Southern Region | |  | |
| 1. What is your highest level of education?  - Less than high school - High school - University level - Higher degrees | | 1. What is your current work status?  - Student - Employed - Unemployed - Retired - Business/trading | |
| 1. What range of income do you have? (SR)  - No income - Below 2000 SAR - 2000 – 4000 SAR - 5000 – 7000 SAR - 8000 – 10000 SAR - Over 10000 SAR | | 1. What is your specialty?  - Medical - Scientific - Literature - No specific field (high school students/education was not completed) | |
| 1. Have you been diagnosed by your doctor with any of the chronic disorders?  - Yes - No | | 1. Do you smoke?  - Yes - No - I am an ex-smoker | |
| 1. How much do you weigh?   ……………. kg | | 1. What is your height?   ……………. cm | |
| **Section 2: Questions related to attitudes and preferences** | | | |
| Are you taking any type of probiotics from foods or supplements?   - Yes - No - Used to take them as supplements | | | |
| **If yes, please answer the following questions:**   - 1. How frequent are you taking pro/prebiotics? - 3-7 times a week - At least once a week - At least once a month   **1.2** What is your main reason for using pro/prebiotics?   - Gastrointestinal discomforts - Immune system issues - Replenish friendly bacteria when taking antibiotics - To maintain health and wellbeing - Other, please specify: ................   1. What is your main influence to try pro/prebiotics? - My own general health knowledge and beliefs - My GP, pharmacist, dietician, nutritionist or other health professionals - Friends and family - Advertisements or social media   1. What is your preferred source of pro/prebiotics? (Tick all that apply) - Natural food sources - Local pharmacy - Online/International pharmacy - No preference   **1.5 In which form are you taking them?** (Tick all that apply)   - Capsule or tablet - Powder - Liquid | **If No, please answer the following questions:**   - 1. Are you willing to use pro/prebiotics in future? - Yes, only if my health professional recommended - Yes, I would be interested in trying - No, I’m not interested in trying   1. What is your main reason for not using pro/prebiotics? - Cost - Side effects - I don’t believe in their effectiveness - I am healthy; I don’t believe I need them - I don’t know about pro/prebiotics - I don’t like supplements   1. If you are going to take pro/prebiotics in the future, what would be your preferred source of pro/prebiotics? - Natural food sources - Supplements - No preference | | **If you used to take them as a supplement, please answer the following questions:**  **3.1** What was your main reason for using pro/prebiotics before?   - Gastrointestinal discomforts - Immune system issues - Replenish friendly bacteria when taking antibiotics - To maintain health and wellbeing - Other, please specify: ................   **3.2** In which form were you taking them? (Tick all that apply)   - Capsule or tablet - Powder - Liquid   **3.3** For how long were you using them?  ……………………………………………   - 1. What was your main reason for stop taking pro/prebiotics? - Cost - Side effects - Low effectiveness - No concerns/issues in consuming these products any more - Others (availability in the market, I don’t like the taste of some products, health condition improved, use them when needed, changed lifestyle, etc.) |
